# Novel compound heterozygous mutations in CNGA1in a Chinese family affected with autosomal recessive retinitis pigmentosa by targeted sequencing

**DOI:** 10.1186/s12886-016-0281-6

**Published:** 2016-07-08

**Authors:** Min Wang, Dekang Gan, Xin Huang, Gezhi Xu

**Affiliations:** Department of Ophthalmology, Eye and ENT Hospital of Fudan University, Shanghai, Chinaᅟ

**Keywords:** CNGA1, Autosomal recessive retinitis pigmentosa, Targeted exome sequencing

## Abstract

**Background:**

About 37 genes have been reported to be involved in autosomal recessive retinitis pigmentosa, a hereditary retinal disease. However, causative genes remain unclear in a lot of cases.

**Methods:**

Two sibs of a Chinese family with ocular disease were diagnosed in Eye and ENT Hospital of Fudan University. Targeted sequencing performed on proband to screen pathogenic mutations. PCR combined Sanger sequencing then performed on eight family members including two affected and six unaffected individuals to determine whether mutations cosegregate with disease.

**Results:**

Two affected members exhibited clinical features that fit the criteria of autosomal recessive retinitis pigmentosa. Two heterozygous mutations (NM000087, p.Y82X and p.L89fs) in CNGA1 were revealed on proband. Affected members were compound heterozygotes for the two mutations whereas unaffected members either had no mutation or were heterozygote carriers for only one of the two mutations. That is, these mutations cosegregate with autosomal recessive retinitis pigmentosa.

**Conclusions:**

Compound heterozygous mutations (NM000087, p.Y82X and p.L89fs) in exon 6 of CNGA1are pathogenic mutations in this Chinese family. Of which, p.Y82X is firstly reported in patient with autosomal recessive retinitis pigmentosa.

**Electronic supplementary material:**

The online version of this article (doi:10.1186/s12886-016-0281-6) contains supplementary material, which is available to authorized users.

## Background

Retinitis pigmentosa (RP, OMIM 268000) refers to a set of hereditary retinal diseases that feature progressive degeneration of the rod and cone photoreceptors. The classic symptom of RP includes early night blindness, progressive increase of the vision field constriction, pigment accumulation in the outer retina and gradually reduced visual acuity. As RP advances, patients will be eventually complete blindness. Nutritional supplementary of vitamin A and docosahexaenoic acid (DHA) in early, could slow progression of disease in many patients [1–4].

RP can be inherited in three patterns-autosomal dominant, autosomal recessive and X-linked which account for 30 ~ 40 %, 50 ~ 60 and 5 ~ 15 % of the RP patients, respectively [5–7]. Digenic inheritance and maternal (mitochondrial) inheritance have also been reported in few cases [8, 9]. By now, 37 genes have been reported to responsible for autosomal recessive retinitis pigmentosa (ARRP) (http://sph.uth.edu/retnet/). Each gene accounts for 0.6 ~ 20 % of ARRP patients. These genes are mainly functioned in following pathways: phototransduction cascade, vitamin A metabolism, structural and cytoskeletal and signaling, cell-cell interaction and synaptic interaction [10]. Although so many genes are identified, causative genes remain unclear in a lot of cases.

Given the harmful of RP, it’s urgent to diagnose as soon as possible. However, RP is highly complicated. It’s difficult to diagnose only rely on clinical features for some cases. Molecular diagnosis is an effective supplementary diagnosis method. The traditional way, PCR combined Sanger sequencing, are time-consuming and costly if there are a lot of candidate genes need to be examined. With the advent of next generation sequencing (NGS) technology, it’s fast to determine the genotype of a large set of genes in parallel. It’s especially useful as the price decrease with the progression of this technology. Target sequencing is a kind of NGS technology which can rapidly capture the entire protein-coding sequence. Numerous causative mutations were accurately and rapidly revealed by this method in recent years [11–14].

In this study, a proband of a Chinese family was subjected to target sequencing and 11 variants are identified as candidate pathogenic mutations. Then, pedigree analysis showed that only two compound heterozygous mutations (NM000087, p.Y82X and p.L89fs) in exon 6 of CNGA1 co-segregate with disease. Of which, p.L89fs was identified in ARRP Japanese patients [15]. P.Y82X is a novel mutation. We conclude that these mutations are responsible for ARRP in this family.

## Methods

### Samples and clinical examination of the RP patients

**Fig. 1:**
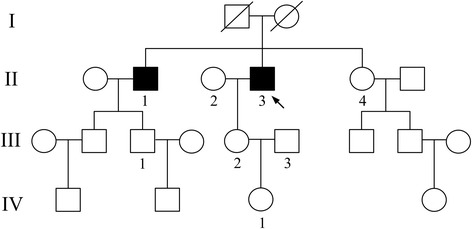
Pedigree of the Chinese family. Circles and squares represent females and males, respectively. Two filled squares represent two patients with ARRP in this family. Proband is indicated by arrow. Subjects who participate in DNA analysis are labeled with numbers

A Chinese family was recruited from Eye and ENT Hospital of Fudan University. The pedigree of this family exhibits ocular disease with a recessive inheritance pattern (Fig. 1). A total of eight members including two affected and six unaffected individuals from this family participated in this study. Before inclusion, complete ophthalmological examinations were carried out on the eight members. These included visual acuity testing (Snellen chart), computerized visual field measurement (Humphrey Visual Field Analyzer, Carl Zeiss Inc., CA, USA), tests of dark adaptation (ECLIPSE Dark Adaptometry), color vision (Ishihara color plate), spectral domain optical coherence tomography (SPECTRALIS® HRA + OCT, Heidelberg Engineering Inc., Heidelberg, Germany) and fundus autofluoresence (SPECTRALIS® HRA + OCT, Heidelberg Engineering Inc., Heidelberg, Germany). Besides, full field electroretinogram (LKC Utas E3000 LKC Technologies Inc., USA) was performed on two affected individuals (II1 and II3, Fig. 1). Medical history of the two affected individuals was also obtained regarding the following aspects: subjective degree of vision loss, age at onset, evolution, medication and other relevant clinical manifestations. In addition, a total of 400 unrelated volunteers from China were served as control group. These volunteers show normal phenotype in visual acuity testing and visual field measurement and don’t have other obvious serious diseases. Blood samples were collected from the eight subjects and stored in 4 °C until analysis.

### DNA isolation and high-throughput sequencing

Genomic DNA was extracted from peripheral blood according to manufacturer’s instructions (QIAGEN, Hilden, Germany). DNA sequencing libraries were then prepared as followed according to Illumina standard protocol: genomic DNA was fragmented; Illumina adapters were ligated to the fragments after ‘A’ ligating to their 3’ends; Fragments with sample size in 200 to 500base pair were selected and amplified by PCR (each sample is tagged with a unique index during this procedure). Fragments in the exonic regions of targeted genes were captured by a specific Hereditary Ophthalmological Disease GenePanel using biotinylatedoligo-probes (MyGenosticsGenCap Enrichment Technologies, MyGenostics, Baltimore, MD, USA). The Panel was designed to detect the coding region of 371 genes which cover almost all of genes that reported to relate to hereditary ophthalmological disease (Additional file 1). The capture experiment was conducted according to the manufacturer’s protocol. Briefly, 1μg DNA library was mixed with Buffer BL and GenCap gene panel probe. The mixture was heated at 95 °C for7min and 65 °C for 2min. Adding 23 μl of the 65 °C prewarmed Buffer HY (MyGenostics) to mixture, then hybridizate at 65 °C for 22 h. After adding 64 μl 2X binding buffer and 80 μl MyOnebeads(Life Technology), the hybrid mixture was transferred to the tube. The mixture was rotated and the beads were washed. The bound DNA was then eluted followed by amplification activated at 98 °C for 30 s (1 cycle), 98 °C for 25 s, 65 °C for 30 s, 72 °C for 30 s (15 cycles), 72 °C for 5 min (1 cycle). The PCR products were purified and then sequenced by IlluminaHiSeq^Tm^ 2000 sequencer, generating 2 × 100 bp reads. Base was called using the Off-Line Base Caller v1.9.

### Bioinformatics analysis

Raw reads were firstly filtered out low quality reads and adaptor sequences using Trimmomatic [16]. The PCR duplicates were removed using the Picard software (http://broadinstitute.github.io/picard/). Then, clean reads were align to the human reference genome (hg19) using BWA [17]. Sequence variations including single nucleotide polymorphisms (SNPs) and insertions or deletions (InDels) were identified using the GATK program (https://www.broadinstitute.org/gatk/). A variant locus with at least five reads support and minor allele frequency >0.3 was kept in variant analysis. The identified SNPs and InDels were annotated using the ANNOVAR (http://annovar.openbioinformatics.org/en/latest/). IGV was used to view the short read alignment and validate the candidate SNPs and InDels (http://www.broadinstitute.org/igv/). The variants are further filtered to identify pathogenic mutations. A variant is determined as pathogenic mutation mainly based on the following three principals: 1) It predicted to alter the sequence of encoded protein, 2) it occurs in different frequency between case group and normal group, 3) it co-segregate with disease in family carrying it. Specifically, novel variants with synonymous effect or minor allele frequency (MAF) >0.01 in 1000 genome variants database or ESP6500 database are discarded. And for known variants, functional effect is not taken into consideration since variant with synonymous effect is yet reported. Only those variants with MAF >0.05 in 1000 genome variants database or ESP6500 database are discarded.

### Mutation analysis of CNGA1

Two mutations in exon 6 of CNGA1 (NM_000087, c.265delC and c.246C > A) were determined by PCR combined with Sanger DNA sequencing in 8 family members. In brief, genomic DNA was extracted and amplified by PCR on standard condition. The primer sequences and precise PCR conditions are available from the authors on request. Undirectional sequencing was carried out using the forward primers. And if this failed, reverse primers were used to sequencing. Sanger sequencing was performed using the ABI PRISMBig Dye Terminator cycle sequencing ready reaction kit on a 3100 ABI DNA sequencer (Thermo-Fisher).

## Results

### Clinical assessments

**Fig. 2:**
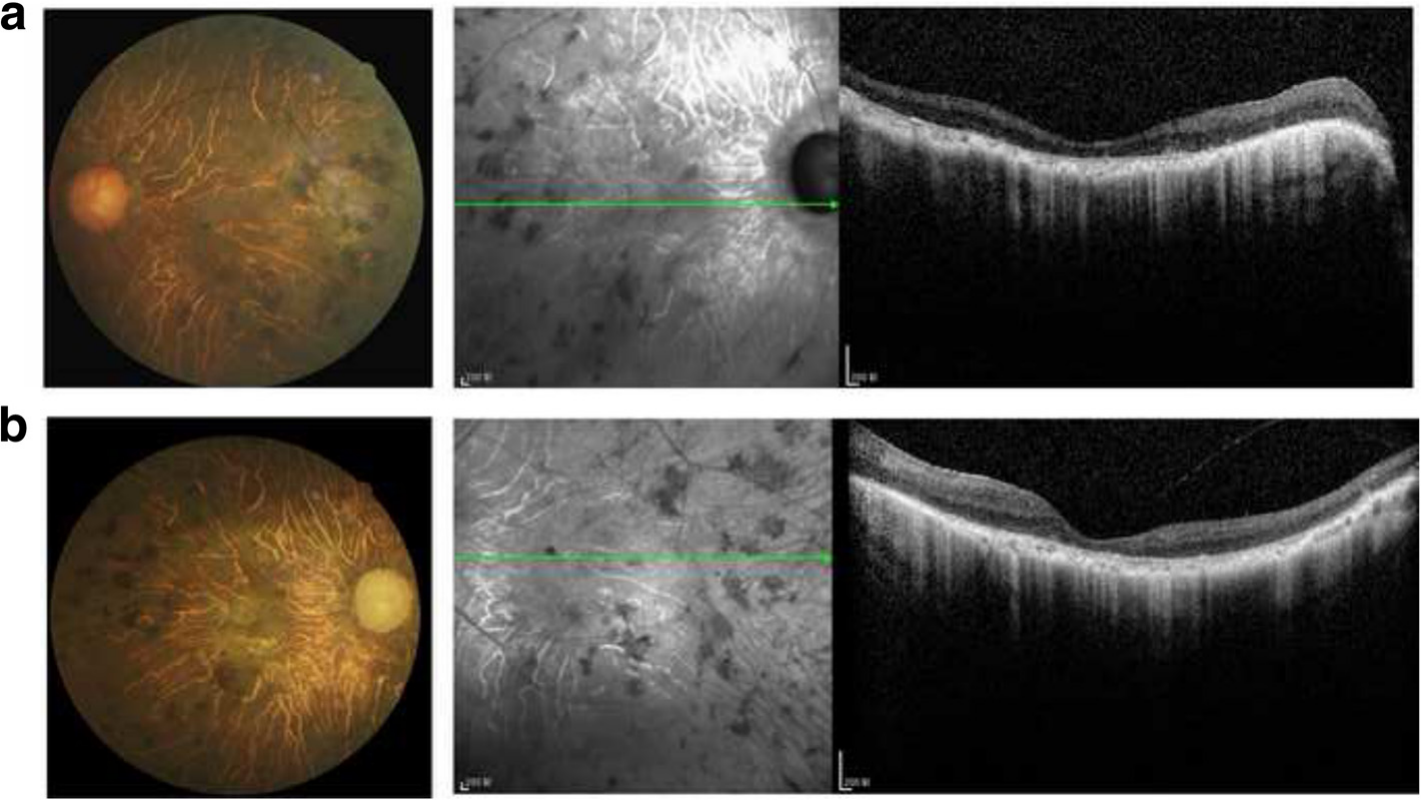
Fundus and OCT images. Fundus and OCT photographs from proband show typical changes of RP. **a**: right eye **b**: left eye

Clinical characteristics of the two affected sibs in this family are listed in Table 1. Both affected individuals suffer from night blindness in early childhood, progressive loss of visual fields and decreased visual acuity with age. All of the features fit the diagnostic criteria of RP. Proband eventually completely lost of his sight when he was ~50. Furthermore, fundus examination of proband shows the typical RP appearance including paravascular bone spicule pigmentation, retinal pigmented epithelium atrophy, pale disc, and arteriolar constriction (Fig. 2). Optical coherence tomography (OCT) showed both eyes of proband had a similar change as shown in Fig. 2. That is, severe ellipsoid band loss in outer retina, obvious loss of retinal nerve fiber, thin retinal pigment epithelium (RPE) and choroid thickness. Meanwhile, both eyes of proband had completely loss of retinal nerve fiber layer (RNFL) without enlargement of cup/disc ratio. The six unaffected individuals showed normal phenotype in all the ophthalmological examinations except that III1 and III2 were suffering from myopia.

**Table 1:** Clinical characteristics of two affected sibs in the family

Patient	II.3	II.1
Onset (years)		
Night blindness	5	9
Visual field defect	16	24
Decreased visual acuity	20	31
Ophthalmological examination		
Visual acuity	RE CF/10	RE 20/60
	LE CF/20	LE 20/80
Slit lamp	Mild cataract	Normal
Visual field	RE/LE: not detectable	RE/LE: nasal and temporal defects
Fundus	RE/LE: paravascular bone spicule pigmentation, RPE atrophy, pale disc, arteriolar constriction	RE/LE: paravascular bone spicule pigmentation, RPE atrophy, pale disc, arteriolar constriction
Optical coherence tomography	RE/LE: extensive ellipsoid band loss, RPE thinning	RE/LE: peripheral ellipsoid band loss, peripheral RPE thinning
Fundus autofluorescence	RE/LE: extensive hypo-fluorescence in the posterior pole	RE/LE: hyper-fluorescent ring in the macula
Electroretinogram	RE/LE: extinguished	RE/LE: extinguished

*RE* right eye, *LE* left eye, *CF* counting finger, *RPE* retinal pigment epithelium

### Pathogenic mutation detection

Targeted sequencing is performed on Proband (II3) using a specific Hereditary Ophthalmological Disease GenePanel. This gene panel includes almost all genes that have been reported to relate to ophthalmological diseases. In total, 5,160, 906 *2 pair-end reads are generated. ~99 % of targeted regions are sequenced, reaching an average depth of ~297 fold which allowed to accurately identification of a SNP. Specifically, the average read depth of ARRP genes is ~311 fold, rang from 56 to 948. A total of 502 variants, including 494 SNVs and eight Indels are identified in target regions. According to the functional effects, the variants are categorized as synonymous, non-synonymous, splice-site, stop-gain, frameshift, non-frameshift and unknown group.

**Fig. 3:**
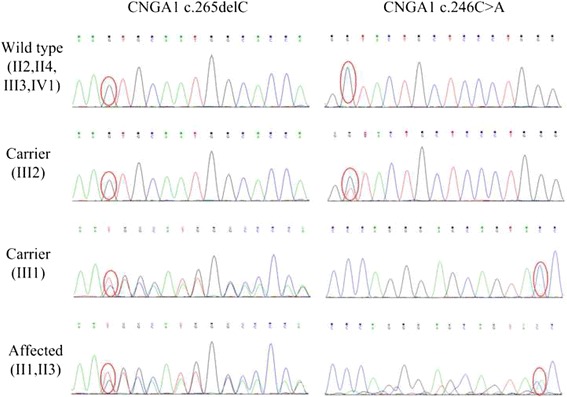
CNGA1 sequence variation analysis. CNGA1 sequence variation pattern are clarified into three types: wild type, no mutation in the two loci; carrier, only one of the two loci in mutant status; affected: two loci were in mutant status. Individuals belong to each type are presented in parenthesis

Eleven variants are identified as candidate pathogenic mutations after filtering step as described in methods. Of which, three variants (ATXN1:NM_000332:p.E357K, TCF4:NM_001243226:c.1793-5G > A and KRT3:NM_057088:c.1189-5T > C) are known pathogenic variants of dominant retinitis pigmentosa. Two variants (CNGA1: NM_000087:Y82X and CNGA1: NM_000087:L89fs) are involved in ARRP. And the rest variants (USH2A:NM_206933:c.11549-5T > -, COL4A3:NM_000091:p.I1567S, TLR4:NM_003266:p.E434K, INPP5E:NM_019892:p.Q633E, HMCN1:NM_031935:p.V170I and KRT6B:NM_005555:p.V454I) are involved in other type of ophthalmological diseases. To determine which variants are the pathogenic mutations, co-segregation analysis is performed on 8 members of this family (II1, II2, II3, II4, III1, III2, III3 and IV1, Fig. 1). Result shows that only the two compound heterozygous mutations in CNGA1co-segregate with this disease (Additional files 2 and 3). Both of the two affected individuals (II1 and II3) carry two heterozygous variants. Two unaffected individuals (III1 and III2) only carry one of the two heterozygous variants. And the other four unaffected individuals (II2, II4, III3 and IV1) are in wild type for the two loci (Fig. 3). Furthermore, the frequency of the two mutations is 0.0074 and 0 in the control group consists of 400 unrelated healthy individuals, respectively (Table 2). Based on mutation analysis and the clinical assessment, we conclude that the compound heterozygous mutations are the pathogenic variants of ARRP in this family.

**Table 2:** CGNA1 mutations detected in proband

Gene symbol	Position	Transcript ID	Exon NO.	DNA change	Protein change	Hom/Het	Frequency
CNGA1	chr4 47951884-47951885	NM_000087	exon 6	c.265delC	p.L89fs	het	0.0074
CNGA1	chr4 47951903	NM_000087	exon 6	c.246C > A	p.Y82X	het	0

*Hom* homozygous, *Het* heterozygous, Frequency: mutation frequency in control group of 400 healthy individuals

CNGA1 was reported as a causative gene of ARRP [18]. The first mutation (CNGA1: NM_000087:L89fs) leads a frame shift from residue 89 of the protein, which was reported in ARRP Japanese patient. And the second one (CNGA1: NM_000087:Y82X), a novel mutation, leads to generate a premature termination codon at residue 82. Both of the two mutations occur early in the reading frame that clearly would change the function of the protein.

## Discussion

CNGA1 encodes a protein-a subunit of the rod cGMP-gated channel, which involved in the phototransduction pathway [19]. Functional protein of CNGA1 consists of six putative transmembrane domains and a pore region. Several mutations in CNGA1 have been revealed to be pathogenic mutations for ARRP by now [15, 18, 20–23]. In which, mutations that lead to deletion of transmembrane domains were reported to be totally nonfunctional. Protein which contains mutations that leads to deletion of C-terminal 32 residues can’t locate to cell membrane although the normal channel activity still maintained. The two mutations (c.265delC and c.246C > A) that we identified in this study occur early in the reading frame which lead to lost most of coding region including all transmembrane domains. It is speculated that no functional CNGA1 protein was generated in affected individuals of this family.

So far, several studies reported RP patients with CNGA1 mutations. All patients including the two sibs we reported here generally showed typical characteristics of RP [15, 20, 24, 25]. For example, they noticed night blindness in early childhood, gradually lost visual field and decreased in visual acuity. Most of them had a severely constrained peripheral visual field. Only one patient showed ring scotoma with a preserved peripheral visual field [15]. In our study, II.1 had a nasal and temporal visual field and II.3 lost all of visual field. Noticeably, II.3 was observed to suffer mild cataract under slit-lamp testing. This was not reported in other patients with CNGA1 mutation. The fundus images of all the patients showed pale optic discs, attenuation of retinal vessels, bone spicule pigmentation and retinal pigment epithelium atrophy. Some of the patients had normal macular region and some of them were identified with retinal degeneration in macular regions. In our study, II.1 showed an abnormal high density ring in macula in the image of fundus autofluorescence which was also observed in a patient from China [24]. Both of the two affected sibs showed extinguished ERG signal which was common in other studies. When scanned by spectral domain optical coherence tomography (SD-OCT), the proband showed unusual retinal changes. That is, both eyes had completely loss of retinal nerve fiber layer (RNFL) without enlargement of cup/disc ratio. This feature was unusual because many RP patients had thicker RNFL even in the late stage of the disease [26]. We assume that this type of genetic mutation may lead to severer optic nerve atrophy than other genotype.

About 2.5 % patients with ARRP are caused by mutations in CNGA1 in western population [25]. Only few studies reported the genetic causes of ARRP in Chinese population [24, 27]. Here we reports mutations in CNGA1 responsible for ARRP in Chinese population, which expand our knowledge about APPR in Chinese population.

## Conclusion

In this study, we descried a Chinese family in which two sibs suffer from severe ocular disease. Proband even completely losses his sight. Disease was transmmitted via autosomal recessive pattern in this family. Clinical measurements show that their symptom fit the criteria of ARRP. By screening 371 genes which have been reported to contribute to hereditary eye disease, two compound heterozygous mutations in the exon 6 of CNGA1 (c.265delC and c.246C > A) were revealed. Furthermore, pedigree analysis was shown that the heterozygous mutations co-segregate with disease. Together, the results demonstrate that the compound heterozygous mutations are the pathogenic mutations of ARRP in this family. Of the two mutations, c.246C > A is a novel mutation.

## Abbreviations

ARRP, Autosomal recessive retinitis pigmentosa; NGS, Next generation sequencing

## Additional files

Additional file 1: Gene symbol list that include in the Hereditary Ophthalmological Disease GenePanel. (XLSX 12 kb) Additional file 2: The status of nine mutations in eight family members which are ruled out from pathogenic mutations. (XLSX 8 kb) Additional file 3: Sequencing image of nine mutations in eight family members which are ruled out from pathogenic mutations. (PDF 159 kb)

## References

[1] Berson EL, Rosner B, Sandberg MA, Hayes KC, Nicholson BW, Weigel-DiFrano C, Willett W (1993). Vitamin A supplementation for retinitis pigmentosa. Arch Ophthalmol.

[2] Sacchetti M, Mantelli F, Merlo D, Lambiase A (2015). Systematic Review of Randomized Clinical Trials on Safety and Efficacy of Pharmacological and Nonpharmacological Treatments for Retinitis Pigmentosa. J Ophthalmol.

[3] DR H m, Locke KG, Wheaton DH, Fish GE, Spencer R, Birch DG (2004). A randomized, placebo-controlled clinical trial of docosahexaenoic acid supplementation for X-linked retinitis pigmentosa. Am J Ophthalmol.

[4] Berson EL, Rosner B, Sandberg MA, Weigel-DiFranco C, Moser A, Brockhurst RJ (2004). Further evaluation of docosahexaenoic acid in patients with retinitis pigmentosa receiving vitamin A treatment: subgroup analyses. Arch Ophthalmol.

[5] Bunker CH, Berson EL, Bromley WC, Hayes RP, Roderick TH (1984). Prevalence of retinitis pigmentosa in Maine. Am J Ophthalmol.

[6] Grondahl J (1987). Estimation of prognosis and prevalence of retinitis pigmentosa and Usher syndrome in Norway. Clin Genet.

[7] Pawlyk BS, Bulgakov OV, Sun X, Adamian M, Shu X, Smith AJ, Berson EL, Ali RR, Khani S, Wright AF, Sandberg MA, Li T (2016). Photoreceptor rescue by an abbreviated human RPGR gene in a murine model of X-linked retinitis pigmentosa. Gene Ther.

[8] Dryja TP, Hahn LB, Kajiwara K, Berson EL (1997). Dominant and digenic mutations in the peripherin/RDS and ROM1 genes in retinitis pigmentosa. Invest Ophthalmol Vis Sci.

[9] Kajiwara K, Berson EL, Dryja TP (1994). Digenic retinitis pigmentosa due tomutations at the unlinked peripherin/RDS and ROM1 loci. Science.

[10] Hartong DT, Berson EL, Dryja TP (2006). Retinitis pigmentosa. Lancet.

[11] Simpson DA, Clark GR, Alexander S, Silvestri G, Willoughby CE (2011). Molecular diagnosis for heterogeneous genetic diseases with targeted high-throughput DNA sequencing applied to retinitis pigmentosa. J Med Genet.

[12] Neveling K, Collin RW, Gilissen C, van Huet RA, Visser L, Kwint MP (2012). Next-generation genetic testing for retinitis pigmentosa. Hum Mutat.

[13] Tucker BA, Scheetz TE, Mullins RF, DeLuca AP, Hoffmann JM, Johnston RM (2011). Exome sequencing and analysis of induced pluripotent stem cells identify the cilia-related gene male germ cell-associated kinase (MAK) as a cause of retinitis pigmentosa. PNAS.

[14] Sun Y, Zhang Z, Cheng J, Lu Y, Yang CL, Luo YY (2015). A novel mutation of EYA4 in a large Chinese family with autosomal dominant middle-frequency sensorineural hearing loss by targeted exome sequencing. J Hum Genet.

[15] Katagiri S, Akahori M, Sergeev Y, Yoshitake K, Ikeo K, Furuno M (2014). Whole exome analysis identifies frequent CNGA1 mutations in Japanese population with autosomal recessive retinitis pigmentosa. PLoS ONE.

[16] Bolger AM, Lohse M, Usadel B (2014). Trimmomatic: A flexible trimmer for Illumina Sequence Data. Bioinformatics.

[17] Li H, Durbin R (2010). Fast and accurate long-read alignment with Burrows-Wheeler Transform. Bioinformatics.

[18] Dryja TP, Finn JT, Peng YW, McGee TL, Berson EL, Yau KW (1995). Mutations in the gene encoding the α subunit of the rod cGMP-gated channel in autosomal recessive retiiitis pigmentosa. Proc Natl Acad Sci.

[19] Yau KW (1994). Phototransduction mechanism in retinal rods and cones. The Friedenwald Lecture. Invest Ophthalmol Vis Sci.

[20] Paloma E, Martínez-Mir A, García-Sandoval B, Ayuso C, Vilageliu L, Gonzàlez-Duarte R (2002). Novel homozygous mutation in the alpha subunit of the rod cGMP gated channel (CNGA1) in two Spanish sibs affected with autosomal recessive retinitis pigmentosa. J Med Genet.

[21] Jin ZB, Mandai M, Yokota T, Higuchi K, Ohmori K, Ohtsuki F (2008). Identifying pathogenic genetic background of simplex or multiplex retinitis pigmentosa patients: a large scale mutation screening study. J Med Genet.

[22] Eisenberger T, Neuhaus C, Khan AO, Decker C, Preising MN, Friedburg C (2013). Increasing the yield in targeted next-generation sequencing by implicating CNV analysis, non-coding exons and the overall variant load: the example of retinal dystrophies. PLoS ONE.

[23] Wang F, Wang H, Tuan HF, Nguyen DH, Sun V, Keser V (2014). Next generation sequencing-based molecular diagnosis of retinitis pigmentosa: identification of a novel genotype-phenotype correlation and clinical refinements. Hum Genet.

[24] Jin X, Qu LH, Hou BK, Xu HW, Meng XH, Pang CP, Yin ZQ (2016). Novel compound heterozygous mutation in the CNGA1 gene underlie autosomal recessive retinitis pigmentosa in a Chinese family. Biosci Rep.

[25] Zhang Q, Zulfiqar F, Riazuddin SA, Xiao X, Ahmad Z, Riazuddin S (2004). Autosomal recessive retinitis pigmentosa in a Pakistani family mapped to CNGA1 with identification of a novel mutation. Mol Vis.

[26] Xue K, Wang M, Chen J, Huang X, Xu G (2013). Retinal nerve fiber layer analysis with scanning laser polarimetry and RTVue-OCT in patients of retinitis pigmentosa. Ophthalmologica.

[27] Xu Y, Guan L, Shen T, Zhang J, Xiao X, Jiang H, Li S, Yang J, Jia X, Yin Y, Guo X, Wang J, Zhang Q (2014). Mutations of 60 known causative genes in 157 families with retinitis pigmentosa based on exome sequencing. Hum Genet.

