# Supplementary material for: Novel compound heterozygous mutations in CNGA1in a Chinese family affected with autosomal recessive retinitis pigmentosa by targeted sequencing
Source: BMC Ophthalmol. 2016 Jul 8;16:101. doi: 10.1186/s12886-016-0281-6 (PMC4938971; doi:10.1186/s12886-016-0281-6)
Supplement: Additional file 3: — Sequencing image of nine mutations in eight family members which are ruled out from pathogenic mutations. (PDF 159 kb) [file 12886_2016_281_MOESM3_ESM.pdf]

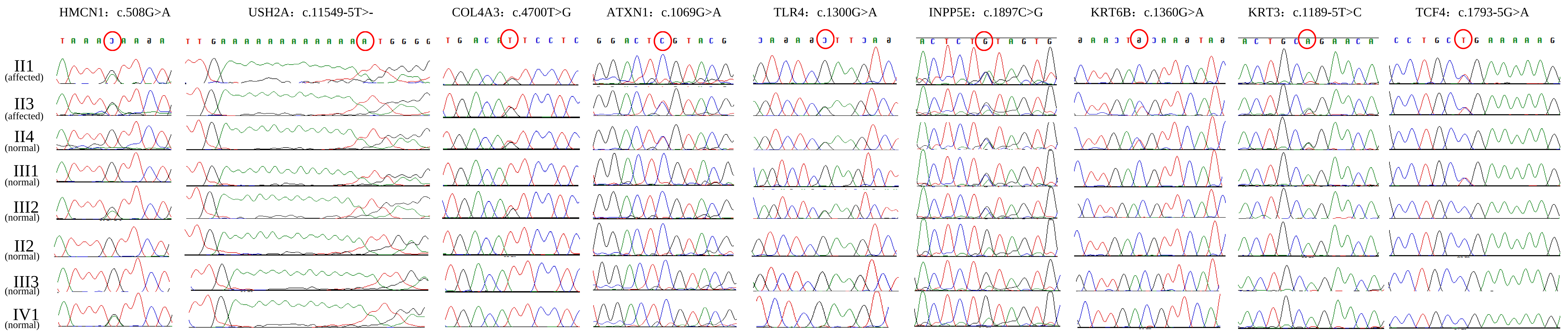
HMCN1：c.508G>A
USH2A：c.11549-5T>-
COL4A3：c.4700T>G
ATXN1：c.1069G>A
TLR4：c.1300G>A
INPP5E：c.1897C>G
KRT6B：c.1360G>A
KRT3：c.1189-5T>C
TCF4：c.1793-5G>A
II1 (affected)
II3 (affected)
II4 (normal)
III1 (normal)
III2 (normal)
II2 (normal)
III3 (normal)
IV1 (normal)
